# Prenatal and postnatal diagnoses and phenotype of 8p23.3p22 duplication in one family

**DOI:** 10.1186/s12920-021-00940-z

**Published:** 2021-03-23

**Authors:** Panlai Shi, Conghui Wang, Yuting Zheng, Xiangdong Kong

**Affiliations:** Genetic and Prenatal Diagnosis Center, Department of Obstetrics and Gynecology, The First Affiliated Hospital of Zhengzhou University, Zhengzhou, 450052 China

**Keywords:** 8p23.1 duplication syndrome, 8p23.3p22 duplication, Phenotype, And heterogeneity

## Abstract

**Background:**

Distal 8p duplication is rare but clinically significant. Duplication syndrome results in variable phenotypes, such as developmental delay, intellectual disability, and malformation of the heart. We aimed to provide a better understanding of the phenotypes by studying duplication and its effects in a single family.

**Methods:**

In a family with a previously induced labor (second fetus) at 12 weeks gestation due to increased nuchal translucency (3.5 mm), copy number variation sequencing (CNV-seq) revealed a 16.22 Mb deletion of 8p23.3p22. For their subsequent pregnancy, the family requested a prenatal diagnosis as well as CNV-seq, karyotyping and FISH testing of all family members.

**Results:**

The first and third children were found to have a 16.22 Mb duplication of 8p23.3p22, containing the 8p23.1 duplication syndrome region. The duplication was inherited from their father, a carrier with a translocation of 8p22 and 22q13. We confirmed that the duplication site was located on chromosome 22q13 by combining the results of CNV-seq, karyotype and FISH. The first child is a 7.5-year-old boy. At one month old, he was diagnosed with a ventricular septal defect and treated surgically at age four. His growth and intelligence developed well, and he performed well in school. His primary issue is an inability to distinguish between the blade alveolars and retroflexes in speech. The third fetus had a normal ultrasound index from beginning until birth. The family elected to continue the pregnancy, and the baby was born healthy, providing us the opportunity to evaluate the effects of 8p23.3p22 duplication by comparison with the brother.

**Conclusion:**

Our study makes a significant contribution to the literature because this relatively rare condition can have significant phenotypical consequences, and an understanding of the inheritance and variability of phenotypes caused by this mutation is essential to an increased understanding of the condition.

## Background

The prevalence of 8p23.1 duplication syndrome is reported as 1 in 58,000 people [1]. 8p23.1 duplication syndrome has a variable phenotype, with three relatively common manifestations: developmental delay, intellectual disability, and malformation of the heart [1–6]. Other phenotypic effects include a wide range of symptoms, such as delayed speech, language development, and an abnormal facial shape. Additionally, it includes behavioral or psychiatric abnormalities [2, 3, 5–7]. The typical genomic region affected includes a 3.68 Mb region; however, a minimal region spanning 776 kb containing only eight genes has been suggested by Barber et al. [1, 8]*.*

The phenotypes vary greatly, from multiple deformities to solely intellectual disabilities. This heterogeneity adds to the challenges of evaluating the condition and providing prenatal genetic counseling to families. The DECIPHER database contains several cases of 8p23.1 duplication. However, only three cases (8:10,001–12,655,629, 12.65 Mb) contain gene duplication regions that are close to and smaller than the region studied in our report (8:160,000–16,380,000, 16.22 Mb). The DECIPHER IDs are 392,337, 395,697, and 399,307 (https://decipher.sanger.ac.uk/). One of these cases was due to a parental unbalanced rearrangement, another was due to maternal inheritance, with the gene being constitutive in the mother, and the cause of the last case was unknown.

Here, we report a 16.22 Mb duplication of 8p23.3p22 in postnatal (the first child, II-1) and prenatal (the third fetus, II-3) members of a family and a deletion in the same region in another member (the second fetus, II-2). The chromosomal abnormality in the three family members resulted from their father, a carrier of a balanced translocation of 8p22 and 22q13.

## Methods

### Molecular cytogenetics

G-banding of chromosomes and fluorescence in situ hybridization (FISH) were carried out using standard techniques. FISH was performed using 8pter (TelVysion 8p Spectrum Green), 8qter (TelVysion 8q Spectrum Orange), and 22qter (TelVysion 22q Spectrum Orange), obtained from the Abbott Laboratories. The microscope used was an Olympus BX53, and the acquisition software used was CytoVision.

### Molecular genetics

Chorionic villus analysis or amniocentesis was performed as previously described [9, 10]. After centrifugation, the cells were washed with PBS, and genomic DNA was extracted using the DNEasy Blood and Tissue Kit (QIAGEN). Quantitative fluorescence PCR (QF-PCR) was used as a quality control procedure to detect DNA contamination. Short tandem repeat (STR) markers were used for chromosome 21, chromosome 18, chromosome 13, and sex chromosomes X and Y according to the process described previously.

Copy number variation sequencing (CNV-seq) was performed as described previously [11]. After BLAST analysis of the original hg19 sequence, CNVs were identified and used to query the following databases: gnomAD (https://gnomad.broadinstitute.org/), DGV (http://dgv.tcag.ca/dgv/app/home), OMIM (https://www.omim.org/), DECIPHER (https://decipher.sanger.ac.uk/), ClinGen (https://dose.https://clinicalgenome.org/) and UCSC (https://genome.ucsc.edu/). Pathogenicity was assessed according to the latest guidelines outlined by the American College of Medical Genetics (ACMG) at five levels: pathogenic, likely pathogenic, variants of uncertain significance, likely benign, and benign.

### Clinical history

**Fig. 1:**
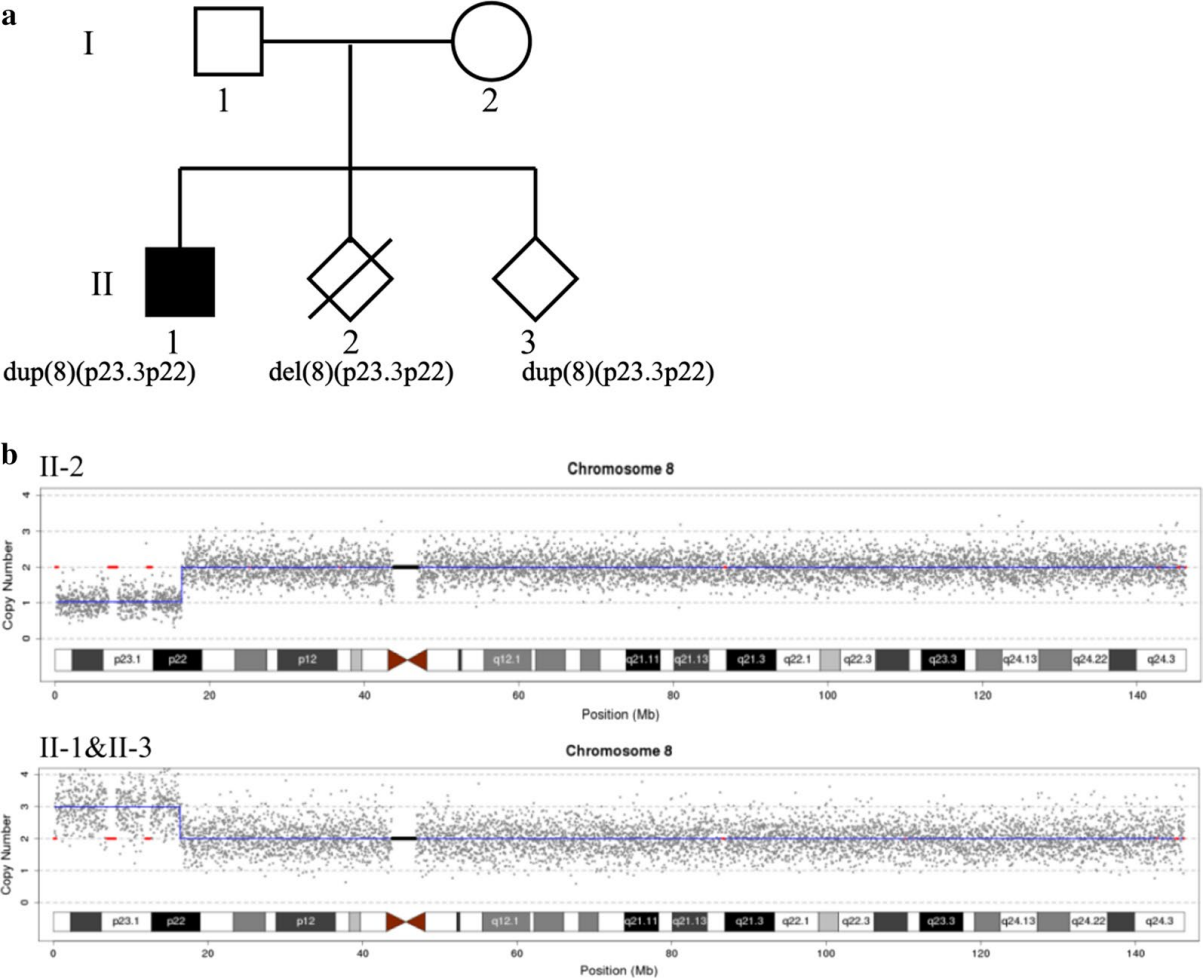
The pedigree of the family (**a**) and the CNV-seq results of II-1, II-2, and II-3 (**b**). The results of the parents were normal and are not shown. The shaded area of II-1 indicates that II-1 has a VSD and cannot distinguish between the blade alveolars and retroflexes in speech

**Fig. 2:**
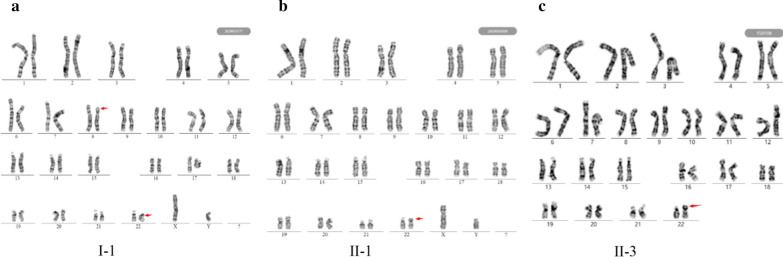
The G-band karyotype of I-1 (**a**), II-1 (**b**), and II-3 (**c**). The result of the mother was normal and is not shown. II-2 was not subjected to G-band karyotyping because the sample was CVS

As shown in Fig. 1, I-2 was a 35-year-old pregnant woman with a history of three pregnancies. Her first child (II-1), a boy, was delivered after a normal pregnancy. At one month of age, he underwent an ultrasound examination that revealed a ventricular septal defect (VSD); he underwent minimally invasive surgery, with a successful outcome, at age four. He is currently 7.5 years old and has normal growth and intellectual development, with a height of 128 cm and a weight of 28.5 kg. However, a slight delay in speech and language development is apparent: he cannot distinguish between the blade alveolars and retroflexes. He did not undergo genetic testing before II-3 received a prenatal diagnosis. The second child (II-2), the fetus, was found to have increased nuchal translucency (3.5 mm) at 12 weeks of gestation and was diagnosed prenatally using chorionic villus sampling (CVS) and CNV-seq testing. The CNV-seq results showed a 16.22 Mb deletion of 8p23.3p22 (seq [hg19] 8p23.3p22 (160,000–16,380,000) × 1), which is associated with a pathogenic CNV that includes the 8p23.1 region. The common phenotypes of 8p23.1 deletion syndrome are a heart abnormality, an atrioventricular canal defect, a congenital diaphragmatic hernia, cryptorchidism, defects in the atrial septum, hyperactivity, and intellectual disability [13–15]. Therefore, the mother elected to terminate the pregnancy following genetic counseling. In her third pregnancy, despite the ultrasound examination being normal, she came to our center for a prenatal diagnosis and family analysis.

## Results

**Fig. 3:**
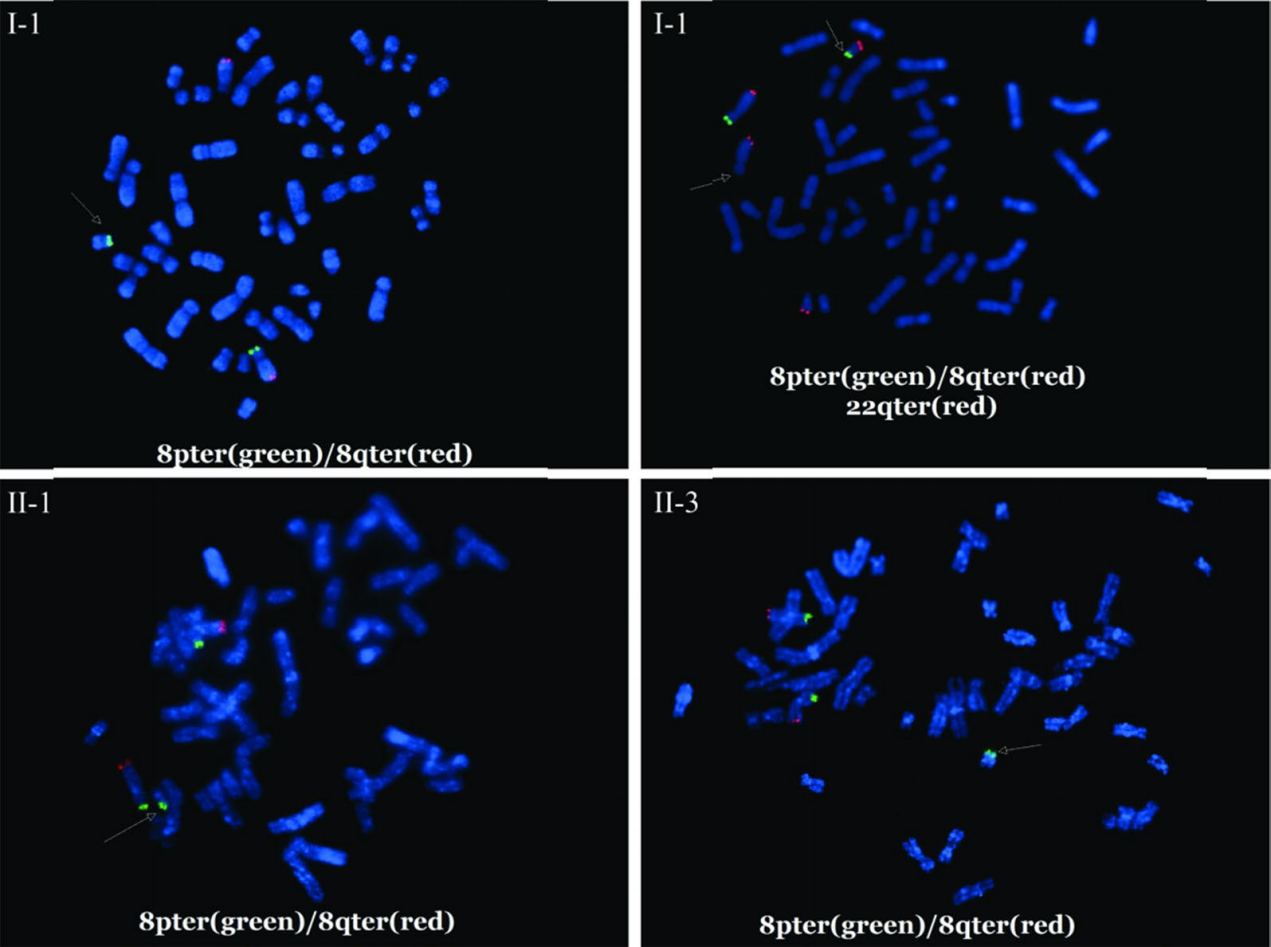
Representative FISH results in I-1, II-1, and II-3

The pedigree of the family is shown in Fig. 1a. The CNV-seq results showed that II-1 and II-3 have the same duplication (seq [hg19]8p23.3p22(160,000–16,380,000) × 3). Individual II-2 had a deletion of 8p23.3p22 (seq [hg19]8p23.3p22(160,000–16,380,000) × 1) (Fig. 1b). The results of both parents were normal (data not shown). banding analysis showed an addition to chromosome 22 in II-1 and II-3 (46,XN,add(22)), and we suspected a balanced translocation of 8p22 and 22p11.2 in the father (I-1, 46,XY,?t(8;22)(p22;p13)). These suspicions were confirmed by FISH (Figs. 2 and 3). The karyotype of the mother was normal (data not shown).

## Discussion

As the father was a carrier of a balanced translocation (t(8;22)(p22;p13)), all three offspring inherited unbalanced chromosome abnormalities: II-1 and II-3 had 8p23.3p22 duplication, and II-2 had 8p23.3p22 deletion. The 16.22 Mb region of 8p23.3p22 contains 81 protein-coding genes, including a large *defensin* cluster (23) and a haploinsufficiency gene, *GATA4*. *GATA4* is related to atrial septal defect 2, atrioventricular septal defect 4, tetralogy of Fallot, and ventricular septal defect 1 in an autosomal dominant manner [OMIM: 600576]. The 8p23.3p22 region is associated with 8p23.1 deletion syndrome and duplication syndrome. Barber et al. reported that 8p23.1 duplication syndrome has a prevalence of 1 in 58,000 and is the reciprocal of 8p23.1 deletion syndrome [1].

The common defects associated with 8p23.1 deletion syndrome are abnormalities of the heart, an atrioventricular canal defect, a congenital diaphragmatic hernia, cryptorchidism, defects in the atrial septum, hyperactivity, and intellectual disability [13–15]. In a prenatal case report, Guimiot et al. showed maternal transmission of an interstitial 8p23.1 deletion of approximately 5.6 Mb to the fetus, which had a normal phenotype according to an ultrasound examination at 20 weeks of gestation; however, the mother displayed moderate intellectual disability and underwent cardiac surgery for a VSD [16]. Faivre et al. described a fetus with a diaphragmatic hernia diagnosed by an ultrasound examination; 8p23.1 deletion was detected at 22 weeks of gestation [17]. In our study, the second fetus (II-2), with 8p23.3p22 deletion, was found to have increased nuchal translucency (3.5 mm) without any other symptoms at 12 weeks of gestation; labor was induced. The heart of the fetus could not be evaluated entirely, possibly due to the early gestational age. The prenatal phenotype of individuals with 8p23.1 deletion, as determined by ultrasound, may vary greatly.

The common symptoms of 8p23.1 duplication syndrome are an abnormal facial shape, behavioral and psychiatric abnormalities, delayed speech and language development, intellectual disability, and malformation of the heart and great vessels [2, 6]. However, the genetic heterogeneity of 8p23.1 duplication syndrome varies considerably. Barber et al. reported four probands with 8p23.1 duplication inherited from their normal parent. The size of the 8p23.1 duplication of the probands ranged from 438 to 802 kb, and the main phenotype was delayed development [1]. The minimal region of overlap was 776 kb, from bases 10,167,881 to 10,943,836. In addition, a considerable number of de novo cases are reported in the DECIPHER database. In the cases of single CNVs, seven (3.05 Mb to 5.24 Mb) were selected (DECIPHER IDs 255,954, 300,950, 322,283, 258,439, 290,136, 262,163, and 356,962) (https://decipher.sanger.ac.uk/search?q=8%3A160000-%2016380000#consented-patients/results). The phenotype of the first five cases included abnormalities of the cardiovascular system, such as VSD or a bicuspid aortic value, abnormalities of the nervous system, including mild global developmental delay, moderate expressive language delay, intellectual disability, and an abnormal facial shape. The other two cases had no clinical phenotypes. Glancy and colleagues reported a duplication of 8p23.1p23.2 between bases 3,539,893 and 10,323,426 associated with speech delay, autism and learning difficulties [18].

Here, we report two cases of a 16.22 Mb duplication of 8p23.3p22 in a family. The first child (II-1) of the family was a 7.5-year-old boy with normal growth and intellectual development; however, he had slightly delayed speech and language development. He underwent successful minimally invasive surgery at age four for VSD. He is currently in the first grade of elementary school and performs well. The genotype of II-3 is the same as that of II-1. No apparent abnormalities were detected in the ultrasound examination of II-3. Due to the heterogeneity of 8p23.1 duplication syndrome and a lack of similarly reported cases, it was difficult to evaluate the possible phenotypes of the fetus. However, after genetic counseling, the mother intended to deliver the child on the expected date, 2020-07-17. During the follow-up, we found that II-3 was born on July 7, 2020 (39^+1^ weeks), weighing 3.5 kg and measuring 50 cm. She is very healthy without any clinical diseases. She is approximately 4 months old with a weight of 7.5 kg and a height of 63 cm. Her growth and development are normal. This situation provides an excellent opportunity for observation of the growth and development of the child after birth.

## Conclusion

We reported a family having a 16.22 Mb duplication of 8p23.3p22 associated with slightly delayed speech and language development in a postnatal fetus (II-1) and in a prenatal fetus (II-3) with a normal ultrasound index. The same region was deleted in II-2; thus, this region was inherited from their father, a carrier of a translocation of 8p22 and 22q13. Our study makes a significant contribution to the literature because this relatively rare condition can have significant phenotypical consequences, and studying the inheritance and variability of phenotypes caused by this mutation is essential to an increased understanding of the condition.

## Data Availability

The raw sequence datasets generated during the current study are not publicly available because it is possible that individual privacy could be compromised. Any questions should be directed to the corresponding author Dr. Xiangdong Kong.

## Abbreviations

CNV: Copy number variation
CNV-seq: CNV sequencing
VSD: Ventricular septal defect
FISH: Fluorescence in situ hybridization

## Declarations

### Content to publication

Written informed consent for publication of clinical details or clinical images was obtained from the parents I-1 and I-2, and the written informed consent for publication of clinical details or clinical images of II-1, II-2, and II-3 were obtained from their parents.
